# Metronidazole and Vancomycin Have a Synergic Effect, with Plant Extracts as Helpful Tools to Combat *Clostridioides difficile* Infections

**DOI:** 10.3390/antibiotics14010054

**Published:** 2025-01-09

**Authors:** Nancy C. Ruiz-Pérez, Yuli Bayona-Pérez, Silvia Laura Guzmán-Gutiérrez, Ricardo Reyes-Chilpa, Víctor M. Luna-Pineda, Javier Torres, Mariana Romo-Castillo

**Affiliations:** 1 División Académica de Ciencias de la Salud, Universidad Juárez Autónoma de Tabasco, Villahermosa 86025, Mexico; nancy_90_116@hotmail.com (N.C.R.-P.); yuli_ewi_bayona@hotmail.com (Y.B.-P.); 2 Colegio de Ciencias y Humanidades, Casa Libertad, Universidad Autónoma de la Ciudad de México, Mexico City 09620, Mexico; saguzmangu@conahcyt.mx; 3 Investigadora e Investigadores por México, Consejo Nacional de Humanidades, Ciencia y Tecnología, Mexico City 03940, Mexico; 4 Instituto de Química, Universidad Nacional Autónoma de México, Mexico City 04510, Mexico; chilpa@unam.mx; 5 Laboratorio de Investigación en Patógenos Respiratorios y Producción de Biológicos, Hospital Infantil de México Federico Gómez, Mexico City 06720, Mexico; luna.pineda@hotmail.com; 6 Unidad de Investigación Médica en EnfermedadesInfecciosas y Parasitarias, Hospital de Pediatría, Centro Médico Nacional Siglo XXI, IMSS, Mexico City 06720, Mexico; uimeip@gmail.com

**Keywords:** *Clostridioides difficile* infection, metronidazole, vancomycin, plant extract, synergic therapy, antibiotic

## Abstract

**Background/Objectives:** The prolonged use of antibiotics is closely related to increased infections caused by *Clostridioides difficile* (Cdiff). Plant-origin compounds have been expanding in recent years as the best opportunity to identify new synergic therapies to combat antibiotic-associated diseases. Mexico has incredible plant biodiversity; natural compounds with antibacterial properties are an alternative treatment. The main objective of this study was to analyze the effect of medicinal plants with an antibacterial action against toxigenic clinical Cdiff strains that have a synergic effect on the antibiotics commonly used to combat this disease. **Methods:** The plants were selected for plants that were previously used in research, and their extracts were tested against Cdiff strains. The antibacterial activity, synergy, and antagonism between the extracts and their synergic effect with antibiotics were evaluated. **Results:** Our results demonstrated that some extracts have effective antimicrobial activity and synergic effects with vancomycin and metronidazole. **Conclusions:** This study suggests that plant extracts and plant compounds derived from these extracts could be used as synergic-antibiotic therapy to combat Cdiff infections.

## 1. Introduction

*Clostridioides difficile* (Cdiff) is responsible for 90 to 100% of cases of pseudomembranous colitis. *C. difficile* infection (CDI) can range from simple colitis to fulminant colitis with fatal consequences. Cdiff is also associated with bacteremia, osteomyelitis, visceral abscesses, and tissue and skin infections [1]. The bacterium’s ability to produce spores resistant to environmental stresses and its capacity to produce potent toxins (Toxin A and Toxin B) make it a persistent and dangerous pathogen in hospital and long-term care settings [2]. The main risk factor in the development of CDI is prolonged therapy with broad-spectrum antibiotics; also, various factors such as prolonged stay in hospitals, immunosuppressed patients or those with chronic diseases, treatment with chemotherapy and radiotherapy, prolonged use of proton pump inhibitors, and invasive gastrointestinal tract procedures are factors that influence the development of CDI [3]. In 2002, with the resurgence of the hypervirulent strain BI/NAP1/027, CDI incidence markedly increased in Europe and North America, increasing this pathogen’s mortality rate [4]. A rise in CDI incidence was reported since 2020 as a product of the COVID-19 pandemic in the USA [5]. Regardless of CDI incidence, the resistance to antibiotics that the new strains present and their high ability to sporulate influence infection recurrence (rCDI) [6]. Cdiff can resist antibiotics through various mechanisms, including the modification of target sites, efflux pump activity, and enzymatic degradation [7]. Cdiff is naturally resistant to beta-lactams due to the lack of the target penicillin-binding proteins in its cell wall structure and aminoglycosides due to the lack of an efficient transport system to bring the drug into the bacterial cell. Cdiff also acquired other resistance, especially to fluoroquinolones, rifampicin, and vancomycin, which made it difficult to treat this infection [7]. In 2014, the European Society of Clinical Microbiology and Infectious Diseases guidelines were published in which metronidazole and vancomycin were the cornerstones of CDI treatment, and in 2021, it was actualized [8], suggesting fidaxomicin as the first-line agent, but its application is more expensive. Other antibiotics, such as nitazoxanide, teicoplanin, and tigecycline, could be an alternative. However, these antibiotics are not recommended as drugs of the first choice due to limited data, patient age, high cost, an unfavorable adverse-event profile, and resistance to Cdiff. For this reason, the search for new treatments or adjuvants for classical therapies is a priority, and traditional medicine could provide us with options.

Plant extracts with antimicrobial properties have gained critical consideration as alternatives to ordinary antimicrobials, particularly in the face of rising antimicrobial resistance [9]. Numerous plants contain bioactive compounds with antimicrobial action, making them essential in conventional pharmaceutical and cutting-edge pharmacology [10]. Using plant-origin components to develop antibiotic therapies to treat CDI could be an effective tool to control the rise of antibiotic resistance and reduce rCDI. A significant number of all drugs on the market are either derived from herbal medicinal plants or inspired by their design. Mexican traditional medicine offers many plants that are used against different gastrointestinal infections [11,12,13,14]. Integrating plant extracts into antibiotic therapy may improve treatment outcomes, reduce the required dosages of antibiotics, and mitigate side effects.

Mexican traditional medicine is a diverse source that has evolved over thousands of years, bridging indigenous knowledge with the influence of other geographical regions like Europe, Africa, and Asia [12]. Plant extracts contain various bioactive compounds that exhibit antibacterial activity against different bacterial pathogens and present several advantages over synthetic antibiotics [15]. One of the principal disadvantages of synthetic antibiotics is the risk of adverse effects such as gastrointestinal disturbances, allergic reactions, or even organ toxicity [16,17]. In contrast, plant extracts are less toxic, with fewer side effects when used in traditional forms [18]. Plant extracts efficiently combat multidrug-resistant bacteria because their complex composition offers a broader spectrum through various mechanisms of action, including cell wall disruption, the inhibition of protein synthesis, DNA interference, and immune modulation. [19,20,21,22,23,24] This approach could lower the risk of resistance development. For this study, 24 plant extracts (PEs) were selected and analyzed for their effective activity against Cdiff. Then, the top six were also analyzed for their synergic activity with vancomycin and metronidazole to identify their potential as synergic antibiotic therapy, which helps combat CDI.

## 2. Results

### 2.1. Inhibition Activity Analysis

Twenty-four extracts were tested (Table 1) against seven clinical MDR Cdiff strains and the reference strain ATCC BAA-1870. By conventional disk diffusion assay, an effective inhibition activity was considered when the PEs had an inhibition ratio of 15 mm for all the strains. Plants were selected based on their traditional application for gastric infection and colitis. Different solvents were used to obtain the extracts, and conventional use selected the part of the plant employed.

**Figure 1:**
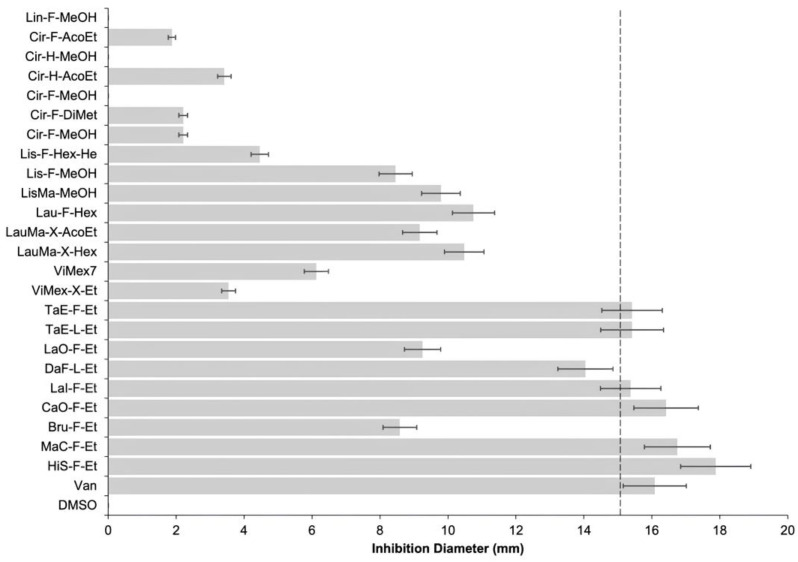
Inhibition zone (mm) of bacterial growth by plant extracts. The mean of the assay of the seven MDR Cdiff strains by triplicate was shown. The line represents the breakpoint resistance for vancomycin (Van) following CLSI recommendations. Nomenclature for all the extracts is shown in Table 1. Dimethyl sulfoxide (DMSO) was employed as the solvent and negative control.

Still, only six had effective antibacterial activity (Figure 1) with an inhibition diameter of >15 mm: HiS-F-Et (Roselle ethanolic flower extract), MaC-F-Et (Chamomile ethanolic flower extract), CaO-F-Et (Marigold ethanolic flower extract), LaOs-F-Et (Lavender etanolic flower extract), TaE-L-Et (Cempasuchil etanolic leaves extract), and TaE-F-Et (Cempasuchil etanolic flower extract). Extracts that did not have effective activity were discarded, and we only worked with the six previously mentioned extracts.

A qualitative evaluation of the six efficient Eps was performed to identify their general composition (Table 2). All the EPs contain flavonoids in different amounts, with Roselle (His-F-Et) having the highest amount and Cempasuchil flower extract (TaE-F-Et) having the lowest. Terpenoids were found only in the Roselle extract (HiS-F-Et) and in Cempsauchil extracts (TaE-L-Et and TaE-F-Et). Alkaloids were found in the Marigold extract (CaO-F-Et) and Cempasuchil extracts. Finally, polyphenols were found in Roselle (His-F-Et), Chamomille (Mac-F-Et), and Marigold (CaO-F-Et). The differential composition and concentration of the active components in the extract suggest that it is possible to have a synergic or antagonist effect between the extracts.

The effective extracts’ minimum inhibitory concentration (MIC) was obtained by dilution, as shown in Table 3. Then, the minimum bactericidal concentration was obtained by inoculating MH agar with the samples from the MIC assay as described. The results show that very low concentrations of the extracts (≥156 μg/mL) are needed to inhibit Cdiff growth; all the extracts have a bactericidal concentration. This suggests that these extracts could be great candidates for creating a therapy against CDI.

### 2.2. The Synergic Effect Between the Extracts

**Figure 2:**
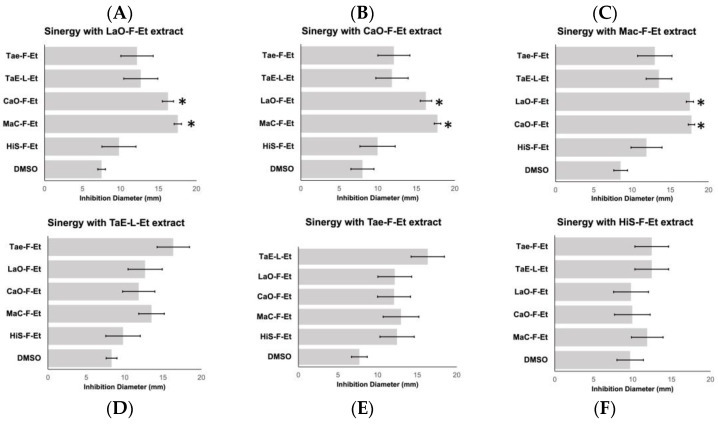
The additive effect between PEs against Cdiff. Mixture 1:1 of the extracts or extract: DMSO was analyzed by disc diffusion. The mean of three independent assays is represented in the graphs, and statistical differential significance (*p* < 0.05) was expressed with an asterisk (*). (**A**). Additive effect with Roselle flower extract (HiS-F-Et). (**B**). Additive effect with Marigold flower extract (Mac-F-Et). (**C**). Additive effect with Chamomile flower extract (CaO-F-Et), (**D**). Additive effect with Lavender flower extract (LaOs-F-Et). (**E**). Additive effect with Cempasuchil leaves extract (TaE-L-Et). (**F**). Additive effect with Cempasuchil flower extract (Tae-F-Et extract).

Growth inhibition by disk diffusion was analyzed using a 1:1 mixture of the extracts to evaluate the possible synergic effect between them. Interestingly, we found a significant positive effect only with three extract mixtures: chamomile/Marigold (MaC-F-Et/CaO-F-E)t, Marigold/Lavender (MaC-F-Et/LaO-F-Et), and Chamomile/Marigold (CaO-F-Et/LaO-F-Et), identifying a significant increase in their activity (Figure 2).

Although disk diffusion identified a statistical difference between some mixtures and the PE inhibition ratio alone, the fractional Inhibitory Concentration Index (FICI) was calculated using the dilution method and sub-MIC concentrations to confirm a synergic effect. The FICI data demonstrate that the mixtures have an indifferent effect because they all have an FIC Index value between 1.10 and 3.71 (Table 4).

### 2.3. The Synergic Effect Between the Extracts and the Antibiotics Against CDI

**Figure 3:**
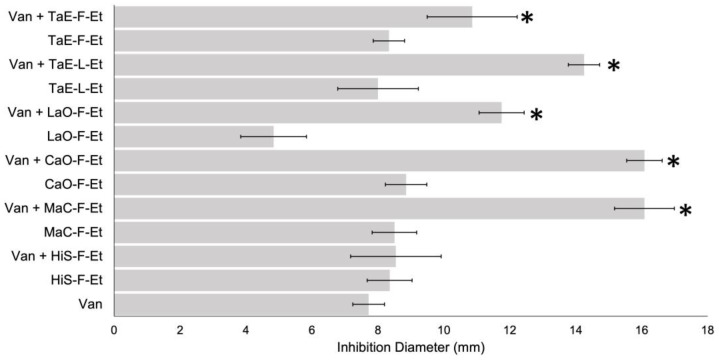
Bacterial inhibition growth of vancomycin mixed with PEs. The disc diffusion method analyzed 1:1 mixtures of PE (1/4 MIC) and vancomycin (30 μg). * *p* < 0.05.

The principal antibiotic therapy against CDI is the application of vancomycin or metronidazole, but the recurrence of CDI is common. These mixtures were analyzed to identify if the EPs have a synergic effect with these antibiotics. First, a growth inhibition assay by disk diffusion was analyzed using a mixture of 250 μg/mL of the PEs with 30 μg of vancomycin. The results shown in Figure 3 demonstrate that vancomycin inhibits the growth cells better in the presence of Chamomile flower (CaO-F-Et), Lavender flower (LaO-F-Et), and Cempasuchitl leaves and flower (TaE-L-Et and TaE-F.Et).

To quantify whether the PEs have a synergistic effect with vancomycin, a checkerboard assay was performed to calculate the FICI (Table 5). A synergic effect was confirmed only with the Van + MaC-F-Et and the Van + CaO-F-Et mixtures, with FICI values of 0.192 and 0.268, respectively. An indifferent impact was observed for the other vancomycin (Van) mixtures, with FICI values of 1.068–2.710.

**Figure 4:**
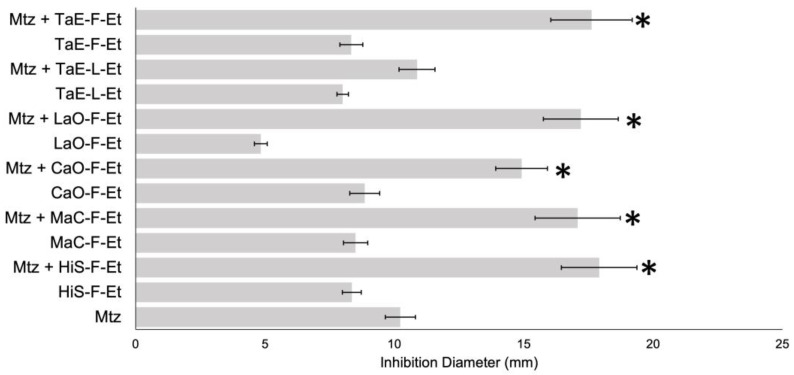
Bacterial inhibition growth of metronidazole mixed with PEs. The disc diffusion method analyzed 1:1 PE (1/4 MIC) and metronidazole (16 ug) mixtures. * *p* < 0.05.

Also, a growth inhibition assay via disk diffusion was performed using a mixture of 250 μg of the PEs with 16 μg of metronidazole. Metronidazole mixed with HiS-F-Et, MaC-F-Et, CaO-F-Et, LaO-F-Et, and TaE-F-Et extracts has better activity than metronidazole alone (Figure 4). Also, FICI was calculated for each mixture, demonstrating that HiS-F-Et, MaC-F-Et, CaO-F-Et, and TaE-F-Et have a synergic effect with metronidazole (Table 5), with values between 0.226 and 0.393. At the same time, LaO-F-Et (FICI = 1.878) and TaE-L-Et (FICI = 3.423) possess an indifferent activity.

## 3. Discussion

*Clostridioides difficile* (Cdiff) is a Gram-positive, spore-forming bacterium that causes antibiotic-associated diarrhea and colitis. Its pathogenicity has increased in recent years due to antibiotic resistance and the ability to form resilient spores. The recurrence of CDI is a problem in controlling this disease, increasing the use of antibiotics and increasing health risks for patients. Recurrent CDI represents a challenge because of the heterogeneity in the symptoms and consequences on patient health. Although many patients respond well to typical medical therapies such as vancomycin and metronidazole, more antibiotics like fidaxomicin are needed. However, the use of fluoroquinolones to control CDI resulted in bacteria resistance. The increase in CDI incidence produced the Centers for Disease Control and Prevention, which classified CDI as an emerging disease that has been an urgent threat since 2019 [27]. The main recommendation includes new strategies to improve CDI and control antibiotic use.

Plant extracts with antibacterial properties offer valuable alternatives for combating bacterial infections, particularly in areas where resistance to conventional antibiotics is becoming more prevalent [28]. Because of their complex composition, plant extracts can act against different bacterial pathways, preventing bacterial resistance, but the phytochemical characteristics of many plants are unknown. Continued research into their mechanisms, efficacy, and applications may pave the way for new therapeutic options in the fight against infectious diseases. This study aimed to identify plant extracts commonly used in Mexican traditional medicine that could effectively control Cdiff and have a synergistic effect with conventional treatments used for CDI control.

Plant extracts have been used for food preservation, pharmaceuticals, and alternative medicine. One of the key aspects of traditional Mexican medicine is using plants or herbal remedies to treat various ailments, including infections. Many of these plants are deeply integrated into the cultural practices of Mexican communities and remain an essential part of modern Mexican healthcare. Many plant-derived compounds have shown potential for combating CDI. For example, garlic contains allicin and other sulfur compounds that have demonstrated antimicrobial activity. In vitro studies have demonstrated that garlic extract can inhibit the growth of Cdiff [29]. Also, curcumin, a turmeric product, possesses anti-clostridial activity and inhibits toxin production and spore formation [30]. In this study, we analyzed the effect of 24 extracts against clinical Cdiff MDR strains. Our results demonstrate that six EPs effectively controlled Cdiff growth: Roselle ethanolic flower extract, Chamomile ethanolic flower extract, Marigold ethanolic flower extract, Lavender ethanolic flower extract, Cempasuchil ethanolic leaves extract, and Cempasuchil ethanolic flower extract. The antibacterial activity of these plants was previously reported against other human pathogens. Antibacterial activity of Roselle (*Hibiscus sabdariffa*) methanolic extract is effective against *S. aureus*, *E. coli*, and *S. typhi* [31], and another study demonstrated that Roselle extracts also could inhibit *Enterococcus faecalis*, a pathogen often associated with urinary tract infections and endodontic infections [32]. *Matricaria chamomilla*, usually called chamomile, has traditionally been used in several countries to cure several diseases, including gastrointestinal disorders, the common cold, liver disorders, and neuropsychiatric and respiratory problems. Chamolie ethanolic flower extract has antibacterial activity against *S. aureus*, *P. aeruginosa*, *Listeria monocytogenes*, *E. faecalis*, *E. coli*, *A. baumanii*, *and K. pneumoniae* [33]. Lavender offers a range of antibacterial benefits, making it a valuable asset in natural healthcare and wellness, and is efficient in controlling *S. aureus*, *S. epidermidis*, *P. aeruginosa*, *E. coli,* and *C. albicans* [34]. *Tagetes erecta* (cempasuchil) has antibacterial activity against *S. aureus*, *E. coli*, *S. epidermidis*, *B. subtilis,* and *B. circulence* [35]. Although the antibacterial activity of these plants was reported against other microorganisms, this study demonstrates the effective antibacterial activity against Cdiff.

A synergistic effect between plant extracts occurs when the combined use of plant extracts is more effective than using the extracts individually. A synergic effect of the extracts was identified only between Chamomile/Marigold (MaC-F-Et/CaO-F-Et), Marigold/Lavender (MaC-F-Et/LaOs-F-Et), and Chamomile/Marigold (CaO-F-Et/LaOs-F-Et), increasing their activity two times.

Many times, using a single antibiotic does not produce the desired effects, and to overcome this, a combination of drugs offers a synergistic effect that becomes more effective. Some of the significant advantages reported for using plant extracts and antibiotics are the increased efficiency, reduction in secondary impacts, increased stability of agents, and dose reduction. Synergism between antimicrobial agents and bioactive plant extracts is a new concept that needs better study. Results showed that Chamomile and Marigold have a synergic effect with vancomycin and metronidazole, while Roselle and Cempasuchil flower extracts have a synergic effect only with metronidazole. This is comparable with other PEs favoring some antibiotic activity, like Tetracycline and erythromycin, with a mango peel (*Mangifera indica*) ethanolic extract against *S. aureus* [36].

This study demonstrates that combining identified medicinal plant extracts with antibiotics offers a new alternative for developing novel antimicrobial therapies and treatments against CDI. Although more studies are needed to understand the mechanism of action of plant extracts and their synergistic mechanism, this direction is essential for identifying new, practical therapies that could be useful for combating emerging diseases like rCDI.

Although this work presents essential advances in the area, it is necessary to consider other factors for correctly implementing these therapies, like standardization techniques needed for clinical evidence and real potential for resistance. Bioactive compounds can vary depending on many factors, such as plant species, growing conditions, and extraction methods, making the standardization of these extracts necessary to ensure consistent therapeutic efficacy. Also, a lack of robust clinical trials demonstrating the safety efficacy of plant-based treatments to combat CDI is required. Finally, further studies are required to identify if the prolonged use of plant-based therapies could lead to the development of resistance in Cdiff.

## 4. Materials and Methods

### 4.1. Microorganisms

*C. difficile* strains were obtained from the UIMEIP of the Pediatric Hospital in the Centro Médico Nacional Siglo XXI in Mexico City. All of them are clinical strains that were previously characterized. Cdiff strains were cultured on pre-reduced Cassman agar supplemented with 5% (*v*/*v*) laked sheep blood and incubated for 24–48 h at 37 °C in anaerobic conditions.

### 4.2. Plant Extracts (PEs)

PEs were selected for previous investigation based on plants that had evidence of use by rural communities in Mexico throughout history. All the extracts are listed in Table 1. They were used at 100 μg/μL to evaluate antimicrobial activity and 250 μg/μL to obtain their MIC and MBC.

Plant material was collected in rural zones and dried at room temperature for two weeks. *Hibiscus sabdariffa* L., *Matricaria chamomilla*, *Brugmansia arborea*, *Calendula officinalis*, *Lavandula x intermedia*, *Datura ferox*, *Lavandula officinalis*, *Tagetes erecta*, and *L. indigo* were obtained from commercial producers from Xochimilco, Mexico City. *Cirsium ehrenberg* was collected in the Cumbres del Ajusco National Park, Mexico City; *Litsea glaucens* were collected from a male in the locality of Huitzila, Veracruz, and *Vismia mexicana* was collected from Xico, Veracruz. The specimens were deposited in the herbarium of the Facultad de Ciencias-UNAM-(FCME) with voucher numbers 163435, 161832, 91674, and 161833. Once dried, plants were crushed and macerated in different solvents for three days. The extracts were filtered and concentrated in a BÜCHIR-II rotary evaporator at reduced pressure, and the remaining solvent was left to dry in an extraction hood.

### 4.3. Evaluation of Antimicrobial Activity of PEs

The antimicrobial effect of the PEs was determined using the Kirby–Bauer disc diffusion technique in anaerobic conditions (90% N_2_, 5% CO_2_ y, 5% H_2_) for 24 h at 37 °C. Next, 100 μL bacterial cultures adjusted to 0.5 McFarland were swab inoculated over Mueller–Hinton agar supplemented with 5% (*v*/*v*) laked sheep blood plates. Then, filter discs containing each 10 μL of PE or antibiotic (5 μg vancomycin or metronidazole) were employed. The negative control plates used dimethyl sulfoxide (DMSO) instead of PEs or antibiotics. The plates were incubated for 48 h at 37 °C in anaerobic conditions. The diameters of the inhibition zones were measured in mm. Each assay was performed in triplicates, and mean values were reported.

The microdilution test determined the PE’s minimum inhibitory concentration (MIC) and the minimum bactericidal concentration (MBC). The dilutions of the PE were 250 to 0.325 μg/mL. Using 96-well plates, 50 μL of each dilution added to 50 μL of MHB was inoculated with 50 μL of bacteria broth adjusted to 0.5 McFarland (106 CFU/mL). After 48 h of incubation at 37 °C in anaerobic conditions, MIC analysis was performed considering the MIC as the concentration without visible growth. Also, 10 μL of each sample was subculture onto Cassman/Blood Agar and incubated for 48 h at 37 °C in anaerobiosis to determine MBC.

### 4.4. Qualitative Evaluation of the EPs

The extracts underwent qualitative phytochemical analysis using standard colorimetric techniques to check for terpenoids, steroids, alkaloids, and polyphenols [18]. Dragendorff tests were performed to identify alkaloids, obtaining red precipitation in positive results. The Salkowski test was employed to test for sterols. The Liebermann–Burchard assay was carried out to detect terpenes. The presence of polyphenols was assessed using the Folin–Ciocalteu test. Initially, 5 mL of each plant extract was evaporated and re-dissolved in 5 mL of chloroform, which was then used for the Dragendorff, Salkowski, and Liebermann–Burchard assays. The Dragendorff test was conducted to identify the presence of alkaloids. An aliquot of 1 mL of the chloroform-resuspended extract was applied to filter paper and treated with Dragendorff reagent; an orange sample indicated a positive result for alkaloids. Plant extracts in chloroform were combined with a single drop of H_2_SO_4_, and a dark brown color indicated a positive outcome for sterols. A combination of 1 mL of the chloroform-resuspended plant extract, five drops of acetic anhydride, and one drop of concentrated H_2_SO_4_ was prepared; if the mixture turned red, it confirmed the presence of terpenes. An ethanolic plant extract was mixed with Folin–Ciocalteu’s reagent (*v*/*v*) and vortexed for 5 min. Subsequently, 1.5 volumes of 20% Na_2_CO_3_ were introduced; if the sample turned blue, it indicated a positive result for polyphenols.

### 4.5. Synergy or Antagonism Effect Between the Extracts

The method used to perform the synergic assay was using E-test probes (Biomérieux, Spain). The synergy between the extracts and antibiotics was determined in Mueller–Hinton/Blood agar in the presence and absence of subinhibitory concentrations of PEs (1/4 of the MIC). As a control, DMSO was added instead of PE. The data were used to calculate the fractional inhibitory concentration (FICI) index and obtain the interaction level between extracts and antibiotics. When the FIC index was ≤0.5, a synergic effect was identified; if it was >0.5 to ≤1, then only an additive effect was reported; if it was from >1.0 to ≤4 the impact between the extracts and the antibiotics was indifferent, and if it was >4 then an antagonism effect was described.

### 4.6. Synergic Effect Between the Extracts and the Antibiotics

The synergic effect between the extracts and the antibiotics was determined by checkerboard in Mueller–Hinton agar as previously described (2), incorporating 10 μL of a 1:1 mixture of both extracts or the extract and the antibiotic (2.5 μg of vancomycin or 1 μg of metronidazole). As a control, DMSO was added instead of the extracts.

### 4.7. Statistical Analysis

The data represent the mean of all strains after three independent replicates. For variance, results were analyzed by One-way ANOVA and Tukey’s multiple range test using ASTATSA software (https://astatsa.com, accessed on 20 October 2024). Differences between means were considered significant at *p*-value < 0.05.

## Figures and Tables

**Table 1:** Plant extracts used in this study against Cdiff.

Nomenclature	Botanical Name	Common Name	Part Plant	Type of Solvent	Reference
HiS-F-Et	*Hibiscus sabdariffa* L.	Roselle	Flower	Ethanol	This study
MaC-F-Et	*Matricaria chamomilla*	Chamomile	Flower	Ethanol	This study
Bru-F-Et	*Brugmansia arborea*	Angel’s trumpet	Flower	Ethanol	This study
CaO-F-Et	*Calendula officinalis*	Marigold	Flower	Ethanol	This study
LaI-F-Et	*Lavandula x intermedia*	Lavandin	Flower	Ethanol	This study
DaF-L-Et	*Datura ferox*	Toloache	Leaves	Ethanol	This study
LaO-F-Et	*Lavandula officinalis*	Lavender	Flower	Ethanol	This study
TaE-L-Et	*Tagetes erecta*	Cempasuchitl	Leaves	Ethanol	This study
TaE-F-Et	*Tagetes erecta*	Cempasuchitl	Flower	Ethanol	This study
ViMex-X-Et	*Vismia mexicana*	Vismia	Aerial	Ethanol	
ViMex7	*Vismia mexicana*	Vismia	Aerial	Ethanol	
LauMa-X-Hex	*Litsea glaucens*	Mexican bay leaf	Aerial	Hexane	
LauMa-X-AcoEt	*Litsea glaucens*	Mexican bay leaf	Leaves	Ethyl Acetate	
Lau-F-Hex	*Litsea glaucens*	Mexican bay leaf	Female Leaves	Ethyl Acetate	
LisMa-MeOH	*Litsea glaucens*	Mexican bay leaf	Male Leaves	Methanol	
Lis-F-MeOH	*Litsea glaucens*	Mexican bay leaf	Female Leaves	Methanol	
Lis-F-Hex-He	*Litsea glaucens*	Mexican bay leaf	Flower	Hexane	
Cir-F-MeOH	*Cirsium ehrenbergil*	Cardo Santo	Flower	Methanol	[25,26]
Cir-F-DiMet	*Cirsium ehrenbergil*	Cardo Santo	Flower	Dichloromethane	
Cir-F-MeOH	*Cirsium ehrenbergil*	Cardo Santo	Flower	Methanol	
Cir-H-AcoEt	*Cirsium ehrenbergil*	Cardo Santo	Leaves	Ethyl Acetate	
Cir-H-MeOH	*Cirsium ehrenbergil*	Cardo Santo	Leaves	Methanol	
Cir-F-AcoEt	*Cirsium ehrenbergil*	Cardo Santo	Flower	Ethyl Acetate	
Lin-F-MeOH	*L. indigo*	Índigo	Flower	Methanol	

**Table 2:** The qualitative composition of the extracts effective against *C. difficile*.

	HiS-F-Et	MaC-F-Et	CaO-F-Et	LaO-F-Et	TaE-L-Et	TaE-F-Et
Flavonoid	+++	++	++	++	++	+
Terpenoid	++	-	-	-	+	++
Steroid	-	-	-	-	-	-
Alkaloid	-	-	+	-	++	+
Polyphenols	+	++	++	-	-	-

**Table 3:** Minimum inhibitory concentration (MIC) and minimum bactericidal concentration (MBC) of efficient plant extracts against Cdiff strains.

	MIC (μg/mL)	MBC (μg/mL)
HiS-F-Et	134.18 ± 8.22	147.04 ± 4.11
MaC-F-Et	155.47 ± 3.02	156.72 ± 1.04
CaO-F-Et	141.03 ± 1.07	159.03 ± 3.07
LaO-F-Et	146.26 ± 4.86	159.03 ± 3.35
TaE-L-Et	152.30 ± 1.90	156.35 ± 1.15
TaE-F-Et	154.71 ± 1.41	167.62 ± 4.85

The mean values of all evaluated strains are represented. HiS-F-Et: Roselle ethanolic flower extract, MaC-F-Et: Chamomile ethanolic flower extract, CaO-F-Et: Marigold ethanolic flower extract, LaO-F-Et: Lavender ethanolic flower extract, TaE-L-Et: Cempasuchil ethanolic leaves extract, TaE-F-Et: Cempasuchil ethanolic flower extract.

**Table 4:** Fractional Inhibitory Concentration Index of PE mixtures.

Extract A	Extract B	FICI index	Extract A	Extract B	FICI Index
HiS-F-Et	MaC-F-Et	1.10	LaOs-F-Et	HiS-F-Et	1.11
	CaO-F-Et	1.06		MaC-F-Et	1.72
	LaOs-F-Et	1.65		CaO-F-Et	1.80
	TaE-L-Et	1.95		TaE-L-Et	1.28
	Tae-F-Et	1.90		Tae-F-Et	1.24
MaC-F-Et	HiS-F-Et	1.79	TaE-L-Et	HiS-F-Et	1.19
	CaO-F-Et	2.70		MaC-F-Et	1.22
	LaOs-F-Et	2.93		CaO-F-Et	1.25
	TaE-L-Et	1.18		LaOs-F-Et	1.31
	Tae-F-Et	1.25		Tae-F-Et	1.39
CaO-F-Et	HiS-F-Et	1.12	Tae-F-Et	HiS-F-Et	1.24
	MaC-F-Et	2.05		MaC-F-Et	1.25
	LaOs-F-Et	1.79		CaO-F-Et	1.22
	TaE-L-Et	1.21		LaOs-F-Et	1.23
	Tae-F-Et	3.71		TaE-L-Et	1.35

FICI < 0.5 is considered a synergic effect, FICI = 0.5–1.0 is an additive effect; when it is between 1.0 and 4.0, it is considered an indifferent effect, and when it is >4.0, it is regarded as an antagonism effect.

**Table 5:** FICI values of the mixture of PEs with vancomycin (Van) and metronidazole (Mtz). FICI of each antibiotic (Ab) mixture with the plant extracts (PEs) was calculated.

Ab/PE Combination	FICI
Van + HiS-F-Et	2.129
Van + MaC-F-Et	0.192
Van + CaO-F-Et	0.268
Van + LaO-F-Et	1.068
Van + TaE-L-Et	1.103
Van + TaE-F-Et	2.710
Mtz + HiS-F-Et	0.393
Mtz + MaC-F-Et	0.340
Mtz + CaO-F-Et	0.226
Mtz + LaO-F-Et	1.878
Mtz + TaE-L-Et	3.423
Mtz + TaE-F-Et	0.392

## Data Availability

Data are contained within the article.
